# Dibromido(6,6′-dimethyl-2,2′-bipyridine-κ^2^ *N*,*N*′)cobalt(II)

**DOI:** 10.1107/S1600536812041980

**Published:** 2012-10-13

**Authors:** Sadif A. Shirvan, Sara Haydari Dezfuli, Fereydoon Khazali, Manouchehr Aghajeri, Ali Borsalani

**Affiliations:** a Department of Chemistry, Omidieh Branch, Islamic Azad University, Omidieh, Iran; b Department of Petroleum Engineering, Omidieh Branch, Islamic Azad University, Omidieh, Iran; c Department of Chemical Engineering, Omidieh Branch, Islamic Azad University, Omidieh, Iran

## Abstract

In the title compound, [CoBr_2_(C_12_H_12_N_2_)], the Co^II^ atom is four-coordinated in a distorted tetra­hedral geometry by two N atoms from a 6,6′-dimethyl-2,2′-bipyridine ligand and by two terminal Br atoms. Inter­molecular C—H⋯Br hydrogen bonds and π–π stacking between the pyridine rings in the *bc* plane [centroid–centroid distance = 3.725 (3) Å] are present in the crystal structure.

## Related literature

For related structures, see: Akbarzadeh Torbati *et al.* (2010 ▶); Alizadeh *et al.* (2011 ▶, 2009 ▶); Itoh *et al.* (2005 ▶); Kou *et al.* (2008 ▶); Onggo *et al.* (2005 ▶); Shirvan & Haydari Dezfuli (2012 ▶).
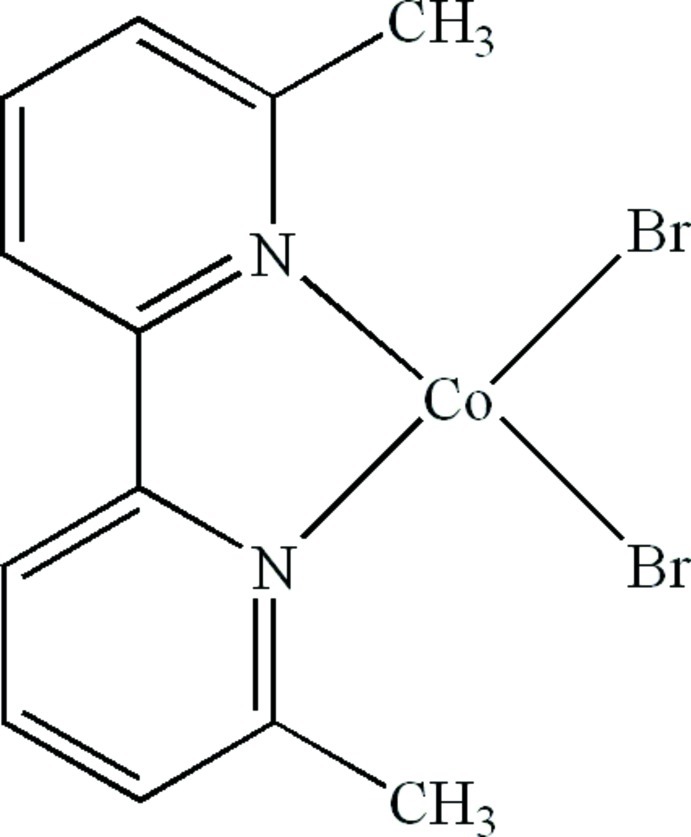


## Experimental

### 

#### Crystal data

[CoBr_2_(C_12_H_12_N_2_)]
*M* *_r_* = 402.97
Monoclinic, 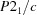

*a* = 7.6550 (6) Å
*b* = 10.2577 (9) Å
*c* = 18.0030 (16) Å
β = 95.779 (7)°
*V* = 1406.5 (2) Å^3^
*Z* = 4
Mo *K*α radiation
μ = 6.88 mm^−1^
*T* = 298 K
0.30 × 0.24 × 0.18 mm

#### Data collection

Bruker APEXII CCD area detector diffractometer
Absorption correction: multi-scan (*SADABS*; Bruker, 2001 ▶) *T*_min_ = 0.149, *T*_max_ = 0.302
7259 measured reflections
2766 independent reflections
1753 reflections with *I* > 2σ(*I*)
*R*_int_ = 0.068

#### Refinement

*R*[*F* ^2^ > 2σ(*F* ^2^)] = 0.045
*wR*(*F* ^2^) = 0.089
*S* = 0.95
2766 reflections
154 parameters
H-atom parameters constrained
Δρ_max_ = 0.51 e Å^−3^
Δρ_min_ = −0.56 e Å^−3^

### 

Data collection: *APEX2* (Bruker, 2005 ▶); cell refinement: *SAINT* (Bruker, 2005 ▶); data reduction: *SAINT*; program(s) used to solve structure: *SHELXTL* (Sheldrick, 2008 ▶); program(s) used to refine structure: *SHELXTL*; molecular graphics: *SHELXTL*; software used to prepare material for publication: *SHELXTL*.

## Supplementary Material

Crystal structure: contains datablock(s) I, global. DOI: 10.1107/S1600536812041980/xu5630sup1.cif

Structure factors: contains datablock(s) I. DOI: 10.1107/S1600536812041980/xu5630Isup2.hkl

Additional supplementary materials:  crystallographic information; 3D view; checkCIF report

## Figures and Tables

**Table 1:** Selected bond lengths (Å)

Co1—N1	2.044 (4)
Co1—N2	2.037 (4)
Co1—Br1	2.3594 (10)
Co1—Br2	2.3588 (10)

**Table 2:** Hydrogen-bond geometry (Å, °)

*D*—H⋯*A*	*D*—H	H⋯*A*	*D*⋯*A*	*D*—H⋯*A*
C8—H8⋯Br1^i^	0.93	2.92	3.696 (5)	142
C12—H12*C*⋯Br1^ii^	0.96	2.89	3.847 (6)	172

Symmetry codes: (i) 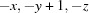; (ii) 
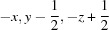
.

**Fig. 1.**
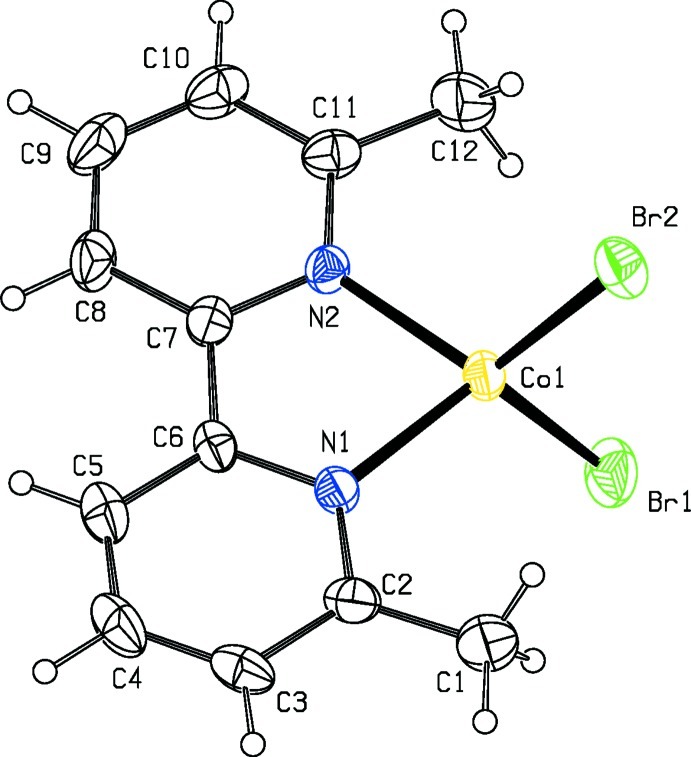
The molecular structure of the title molecule, with the atom-numbering scheme. Displacement ellipsoids are drawn at the 30% probability level.

**Fig. 2.**
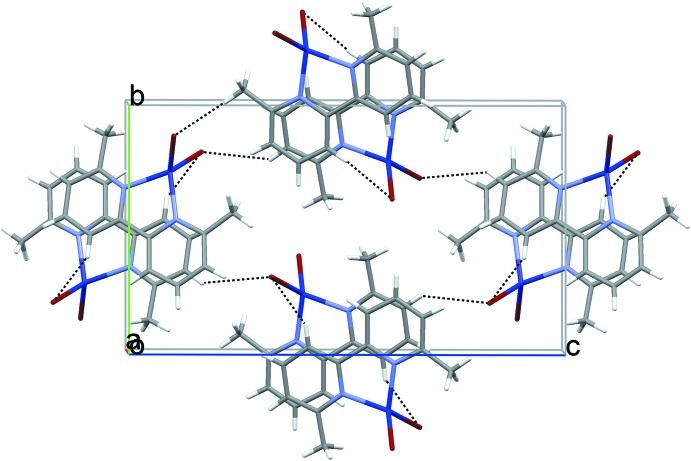
Unit-cell packing diagram for title molecule. Hydrogen bonds are shown as dashed lines.

## supplementary crystallographic information

### Comment

6,6'-Dimethyl-2,2'-bipyridine (6,6'-dmbipy), is a good bidentate ligand, and numerous complexes with 6,6'-dmbipy have been prepared, such as that of zinc (Alizadeh *et al.*, 2009), copper (Itoh *et al.*, 2005), cadmium (Shirvan & Haydari Dezfuli, 2012), cobalt (Akbarzadeh Torbati *et al.*, 2010), nickel (Kou *et al.*, 2008), ruthenium (Onggo *et al.*, 2005) and mercury (Alizadeh *et al.*, 2011). We report herein the synthesis and crystal structure of the title compound.

In the title compound, (Fig. 1), the Co^II^ atom is four-coordinated in a distorted tetrahedral geometry by two N atoms from a 6,6'-dimethyl-2,2'-bipyridine and two terminal Br atoms. The Co—Br and Co—N bond lengths and angles are collected in Table 1.

In the crystal structure, intermolecular C—H···Br hydrogen bonds (Table 2) and π-π contacts (Fig. 2) between the pyridine rings, *Cg*2—*Cg*3^i^ [symmetry cods: (i) -*x*, 1 - y, -*z*, where *Cg*2 and *Cg*3 are centroids of the rings (N1/C2–C6) and (N2/C7–C11), respectively] may stabilize the structure, with centroid–centroid distance of 3.725 (3) Å.

### Experimental

For the preparation of the title compound, a solution of 6,6'-dimethyl-2,2'-bipyridine (0.25 g, 1.33 mmol) in methanol (10 ml) was added to a solution of CoBr_2_ (0.29 g, 1.33 mmol) in acetonitrile (15 ml) and the resulting blue solution was stirred for 15 min at 313 K. This solution was left to evaporate slowly at room temperature. After one week, blue block crystals of the title compound were isolated (yield 0.41 g, 76.5%).

### Refinement

H atoms were positioned geometrically with C—H = 0.93 and 0.96 Å and constrained to ride on their parent atoms, *U*_iso_(H) = 1.2*U*_eq_(C) for methyl H atoms and 1.2*U*_eq_(C) for aromatic H atoms.

### Crystal data

**Table:**

[CoBr_2_(C_12_H_12_N_2_)]	*F*(000) = 780
*M**_r_* = 402.97	*D*_x_ = 1.903 Mg m^−^^3^
Monoclinic, *P*2_1_/*c*	Mo *K*α radiation, λ = 0.71073 Å
Hall symbol: -P 2ybc	Cell parameters from 7259 reflections
*a* = 7.6550 (6) Å	θ = 2.3–26.0°
*b* = 10.2577 (9) Å	µ = 6.88 mm^−^^1^
*c* = 18.0030 (16) Å	*T* = 298 K
β = 95.779 (7)°	Block, blue
*V* = 1406.5 (2) Å^3^	0.30 × 0.24 × 0.18 mm
*Z* = 4	

### Data collection

**Table:**

Bruker APEXII CCD area detector diffractometer	2766 independent reflections
Radiation source: fine-focus sealed tube	1753 reflections with *I* > 2σ(*I*)
Graphite monochromator	*R*_int_ = 0.068
ω scans	θ_max_ = 26.0°, θ_min_ = 2.3°
Absorption correction: multi-scan (*SADABS*; Bruker, 2001)	*h* = −8→9
*T*_min_ = 0.149, *T*_max_ = 0.302	*k* = −12→11
7259 measured reflections	*l* = −22→22

### Refinement

**Table:**

Refinement on *F*^2^	Primary atom site location: structure-invariant direct methods
Least-squares matrix: full	Secondary atom site location: difference Fourier map
*R*[*F*^2^ > 2σ(*F*^2^)] = 0.045	Hydrogen site location: inferred from neighbouring sites
*wR*(*F*^2^) = 0.089	H-atom parameters constrained
*S* = 0.95	*w* = 1/[σ^2^(*F*_o_^2^) + (0.0371*P*)^2^] where *P* = (*F*_o_^2^ + 2*F*_c_^2^)/3
2766 reflections	(Δ/σ)_max_ = 0.005
154 parameters	Δρ_max_ = 0.51 e Å^−^^3^
0 restraints	Δρ_min_ = −0.56 e Å^−^^3^

### Special details

**Table:**

Geometry. All e.s.d.'s (except the e.s.d. in the dihedral angle between two l.s. planes) are estimated using the full covariance matrix. The cell e.s.d.'s are taken into account individually in the estimation of e.s.d.'s in distances, angles and torsion angles; correlations between e.s.d.'s in cell parameters are only used when they are defined by crystal symmetry. An approximate (isotropic) treatment of cell e.s.d.'s is used for estimating e.s.d.'s involving l.s. planes.
Refinement. Refinement of *F*^2^ against ALL reflections. The weighted *R*-factor *wR* and goodness of fit *S* are based on *F*^2^, conventional *R*-factors *R* are based on *F*, with *F* set to zero for negative *F*^2^. The threshold expression of *F*^2^ > σ(*F*^2^) is used only for calculating *R*-factors(gt) *etc*. and is not relevant to the choice of reflections for refinement. *R*-factors based on *F*^2^ are statistically about twice as large as those based on *F*, and *R*- factors based on ALL data will be even larger.

### Fractional atomic coordinates and isotropic or equivalent isotropic displacement parameters (Å^2^)

**Table:**

	*x*	*y*	*z*	*U*_iso_*/*U*_eq_	
C1	0.3959 (11)	0.8727 (6)	−0.0503 (3)	0.074 (2)	
H1A	0.4872	0.8803	−0.0098	0.089*	
H1B	0.2921	0.9159	−0.0372	0.089*	
H1C	0.4338	0.9122	−0.0942	0.089*	
C2	0.3572 (8)	0.7343 (6)	−0.0648 (3)	0.0494 (14)	
C3	0.3757 (8)	0.6766 (7)	−0.1338 (3)	0.0566 (16)	
H3	0.4156	0.7252	−0.1723	0.068*	
C4	0.3342 (9)	0.5468 (7)	−0.1441 (3)	0.0651 (18)	
H4	0.3435	0.5077	−0.1901	0.078*	
C5	0.2791 (8)	0.4753 (6)	−0.0865 (3)	0.0550 (15)	
H5	0.2506	0.3876	−0.0930	0.066*	
C6	0.2665 (7)	0.5352 (5)	−0.0188 (3)	0.0397 (12)	
C7	0.2137 (7)	0.4644 (5)	0.0477 (3)	0.0421 (12)	
C8	0.1766 (8)	0.3330 (5)	0.0476 (3)	0.0570 (16)	
H8	0.1821	0.2838	0.0045	0.068*	
C9	0.1312 (9)	0.2751 (6)	0.1121 (4)	0.0687 (19)	
H9	0.1056	0.1865	0.1131	0.082*	
C10	0.1245 (8)	0.3503 (6)	0.1744 (4)	0.0592 (16)	
H10	0.0960	0.3120	0.2185	0.071*	
C11	0.1592 (8)	0.4813 (6)	0.1731 (3)	0.0504 (14)	
C12	0.1535 (10)	0.5666 (7)	0.2391 (3)	0.077 (2)	
H12A	0.0704	0.6355	0.2275	0.092*	
H12B	0.2677	0.6033	0.2525	0.092*	
H12C	0.1187	0.5164	0.2801	0.092*	
N1	0.3039 (6)	0.6627 (4)	−0.0087 (2)	0.0385 (10)	
N2	0.2047 (6)	0.5381 (4)	0.1095 (2)	0.0387 (10)	
Co1	0.26905 (10)	0.72871 (7)	0.09589 (3)	0.0414 (2)	
Br1	0.03425 (10)	0.87570 (7)	0.10302 (4)	0.0703 (2)	
Br2	0.52149 (9)	0.80174 (6)	0.17019 (3)	0.0598 (2)	

### Atomic displacement parameters (Å^2^)

**Table:**

	*U*^11^	*U*^22^	*U*^33^	*U*^12^	*U*^13^	*U*^23^
C1	0.109 (6)	0.062 (4)	0.054 (3)	−0.021 (4)	0.019 (4)	0.010 (3)
C2	0.053 (4)	0.056 (3)	0.039 (3)	0.008 (3)	0.002 (3)	0.006 (2)
C3	0.057 (4)	0.082 (5)	0.032 (3)	0.006 (3)	0.009 (3)	0.003 (3)
C4	0.067 (5)	0.085 (5)	0.043 (3)	0.013 (4)	0.007 (3)	−0.025 (3)
C5	0.056 (4)	0.058 (4)	0.051 (3)	0.002 (3)	0.005 (3)	−0.019 (3)
C6	0.034 (3)	0.043 (3)	0.040 (3)	0.003 (2)	−0.001 (2)	−0.011 (2)
C7	0.035 (3)	0.037 (3)	0.051 (3)	0.002 (2)	−0.007 (2)	−0.003 (2)
C8	0.056 (4)	0.041 (3)	0.070 (4)	−0.008 (3)	−0.012 (3)	−0.012 (3)
C9	0.066 (5)	0.038 (3)	0.099 (5)	−0.010 (3)	−0.010 (4)	0.012 (3)
C10	0.054 (4)	0.051 (4)	0.072 (4)	−0.013 (3)	0.001 (3)	0.018 (3)
C11	0.046 (4)	0.053 (4)	0.052 (3)	−0.005 (3)	0.006 (3)	0.011 (3)
C12	0.112 (6)	0.076 (5)	0.047 (3)	−0.018 (4)	0.027 (4)	0.006 (3)
N1	0.042 (3)	0.039 (2)	0.035 (2)	−0.002 (2)	0.0043 (19)	−0.0038 (17)
N2	0.038 (3)	0.034 (2)	0.043 (2)	−0.005 (2)	0.0007 (19)	−0.0013 (18)
Co1	0.0525 (5)	0.0357 (4)	0.0369 (3)	−0.0046 (3)	0.0083 (3)	−0.0058 (3)
Br1	0.0725 (5)	0.0601 (4)	0.0781 (4)	0.0177 (4)	0.0062 (3)	−0.0207 (3)
Br2	0.0619 (4)	0.0660 (4)	0.0508 (3)	−0.0135 (3)	0.0023 (3)	−0.0131 (3)

### Geometric parameters (Å, º)

**Table:**

C1—C2	1.468 (8)	C8—C9	1.379 (8)
C1—H1A	0.9600	C8—H8	0.9300
C1—H1B	0.9600	C9—C10	1.367 (9)
C1—H1C	0.9600	C9—H9	0.9300
C2—N1	1.345 (6)	C10—C11	1.370 (8)
C2—C3	1.396 (7)	C10—H10	0.9300
C3—C4	1.377 (9)	C11—N2	1.360 (6)
C3—H3	0.9300	C11—C12	1.481 (8)
C4—C5	1.371 (9)	C12—H12A	0.9600
C4—H4	0.9300	C12—H12B	0.9600
C5—C6	1.378 (7)	C12—H12C	0.9600
C5—H5	0.9300	Co1—N1	2.044 (4)
C6—N1	1.347 (6)	Co1—N2	2.037 (4)
C6—C7	1.490 (7)	Co1—Br1	2.3594 (10)
C7—N2	1.354 (6)	Co1—Br2	2.3588 (10)
C7—C8	1.377 (7)		
			
C2—C1—H1A	109.5	C10—C9—C8	118.8 (6)
C2—C1—H1B	109.5	C10—C9—H9	120.6
H1A—C1—H1B	109.5	C8—C9—H9	120.6
C2—C1—H1C	109.5	C9—C10—C11	121.0 (6)
H1A—C1—H1C	109.5	C9—C10—H10	119.5
H1B—C1—H1C	109.5	C11—C10—H10	119.5
N1—C2—C3	120.1 (5)	N2—C11—C10	120.1 (5)
N1—C2—C1	117.7 (5)	N2—C11—C12	116.9 (5)
C3—C2—C1	122.1 (5)	C10—C11—C12	122.9 (5)
C4—C3—C2	119.2 (5)	C11—C12—H12A	109.5
C4—C3—H3	120.4	C11—C12—H12B	109.5
C2—C3—H3	120.4	H12A—C12—H12B	109.5
C5—C4—C3	120.0 (5)	C11—C12—H12C	109.5
C5—C4—H4	120.0	H12A—C12—H12C	109.5
C3—C4—H4	120.0	H12B—C12—H12C	109.5
C4—C5—C6	119.0 (6)	C2—N1—C6	120.4 (4)
C4—C5—H5	120.5	C2—N1—Co1	126.0 (3)
C6—C5—H5	120.5	C6—N1—Co1	113.5 (3)
N1—C6—C5	121.3 (5)	C7—N2—C11	119.4 (4)
N1—C6—C7	115.9 (4)	C7—N2—Co1	113.8 (3)
C5—C6—C7	122.8 (5)	C11—N2—Co1	126.8 (3)
N2—C7—C8	121.3 (5)	N2—Co1—N1	81.30 (15)
N2—C7—C6	115.4 (4)	N2—Co1—Br2	115.50 (12)
C8—C7—C6	123.3 (5)	N1—Co1—Br2	116.88 (13)
C7—C8—C9	119.4 (6)	N2—Co1—Br1	114.33 (13)
C7—C8—H8	120.3	N1—Co1—Br1	115.65 (12)
C9—C8—H8	120.3	Br2—Co1—Br1	110.57 (4)
			
N1—C2—C3—C4	1.8 (9)	C5—C6—N1—Co1	178.5 (4)
C1—C2—C3—C4	−178.9 (6)	C7—C6—N1—Co1	−2.2 (6)
C2—C3—C4—C5	−1.4 (10)	C8—C7—N2—C11	0.4 (8)
C3—C4—C5—C6	−0.1 (10)	C6—C7—N2—C11	−179.4 (5)
C4—C5—C6—N1	1.3 (9)	C8—C7—N2—Co1	179.1 (5)
C4—C5—C6—C7	−177.9 (5)	C6—C7—N2—Co1	−0.6 (6)
N1—C6—C7—N2	1.9 (7)	C10—C11—N2—C7	0.6 (8)
C5—C6—C7—N2	−178.8 (5)	C12—C11—N2—C7	179.4 (5)
N1—C6—C7—C8	−177.9 (5)	C10—C11—N2—Co1	−177.9 (4)
C5—C6—C7—C8	1.4 (9)	C12—C11—N2—Co1	0.8 (8)
N2—C7—C8—C9	−0.7 (9)	C7—N2—Co1—N1	−0.4 (4)
C6—C7—C8—C9	179.0 (5)	C11—N2—Co1—N1	178.2 (5)
C7—C8—C9—C10	0.0 (10)	C7—N2—Co1—Br2	−116.1 (3)
C8—C9—C10—C11	1.1 (10)	C11—N2—Co1—Br2	62.5 (5)
C9—C10—C11—N2	−1.4 (10)	C7—N2—Co1—Br1	113.9 (3)
C9—C10—C11—C12	180.0 (6)	C11—N2—Co1—Br1	−67.5 (5)
C3—C2—N1—C6	−0.6 (8)	C2—N1—Co1—N2	−179.1 (5)
C1—C2—N1—C6	−180.0 (6)	C6—N1—Co1—N2	1.5 (4)
C3—C2—N1—Co1	180.0 (4)	C2—N1—Co1—Br2	−64.8 (5)
C1—C2—N1—Co1	0.6 (8)	C6—N1—Co1—Br2	115.7 (3)
C5—C6—N1—C2	−0.9 (8)	C2—N1—Co1—Br1	68.0 (5)
C7—C6—N1—C2	178.3 (5)	C6—N1—Co1—Br1	−111.4 (3)

### Hydrogen-bond geometry (Å, º)

**Table:**

*D*—H···*A*	*D*—H	H···*A*	*D*···*A*	*D*—H···*A*
C8—H8···Br1^i^	0.93	2.92	3.696 (5)	142
C12—H12*C*···Br1^ii^	0.96	2.89	3.847 (6)	172

Symmetry codes: (i) −*x*, −*y*+1, −*z*; (ii) −*x*, *y*−1/2, −*z*+1/2.
